# Supplementary material for: Harnessing Nuclear Energy to Gold Nanoparticles for the Concurrent Chemoradiotherapy of Glioblastoma
Source: Nanomaterials (Basel). 2023 Oct 24;13(21):2821. doi: 10.3390/nano13212821 (PMC10650840; doi:10.3390/nano13212821)
Supplement: Supplementary file 1 [file nanomaterials-13-02821-s001.zip › nanomaterials-2341445-supplementary.pdf]

Supplementary Information

Harnessing Nuclear Energy to Gold Nanoparticles for the Concurrent Chemoradiotherapy of Glioblastoma

Jui-Ping Li [1,†], Yu-Cheng Kuo [2,3,†], Wei-Neng Liao [1], Ya-Ting Yang [1], Sih-Yu Chen [1], Yu-Ting Chien [1],
Kuo-Hung Wu [4], Mei-Ya Wang [4], Fong-In Chou [4], Mo-Hsiung Yang [5], Dueng-Yuan Hueng [6], Chung-Shi Yang [1]
and Jen-Kun Chen [1,7,8,*]

[1] Institute of Biomedical Engineering and Nanomedicine, National Health Research Institutes,
Miaoli 35053, Taiwan; piny72@nhri.edu.tw (J.-P.L.); 970730@nhri.edu.tw (W.-N.L.);
fructose0129@hotmail.com (Y.-T.Y.); sihyu2011@gmail.com (S.-Y.C.);
ken800322@gmail.com (Y.-T.C.); cyang@nhri.edu.tw (C.-S.Y.)
[2] Department of Radiation Oncology, China Medical University Hospital, Taichung 40447, Taiwan; shapico22@gmail.com
[3] School of Medicine, China Medical University, Taichung 40402, Taiwan
[4] Nuclear Science and Technology Development Center, National Tsing Hua University,
Hsinchu 30013, Taiwan; khwu@mx.nthu.edu.tw (K.-H.W.); meywang@mx.nthu.edu.tw (M.-Y.W.);
fichou@mx.nthu.edu.tw (F.-I.C.)
[5] Department of Biomedical Engineering and Environmental Sciences, National Tsing Hua University,
Hsinchu 30013, Taiwan; mhyang@mx.nthu.edu.tw
[6] School of Medicine, National Defense Medical Center, Taipei 11490, Taiwan; hondy2195@yahoo.com.tw
[7] Biotechnology Center, National Chung Hsing University, Taichung 40227, Taiwan
[8] Graduate Institute of Life Sciences, National Defense Medical Center, Taipei 11490, Taiwan
* Correspondence: jkchen@nhri.edu.tw
† These authors contributed equally to this work.

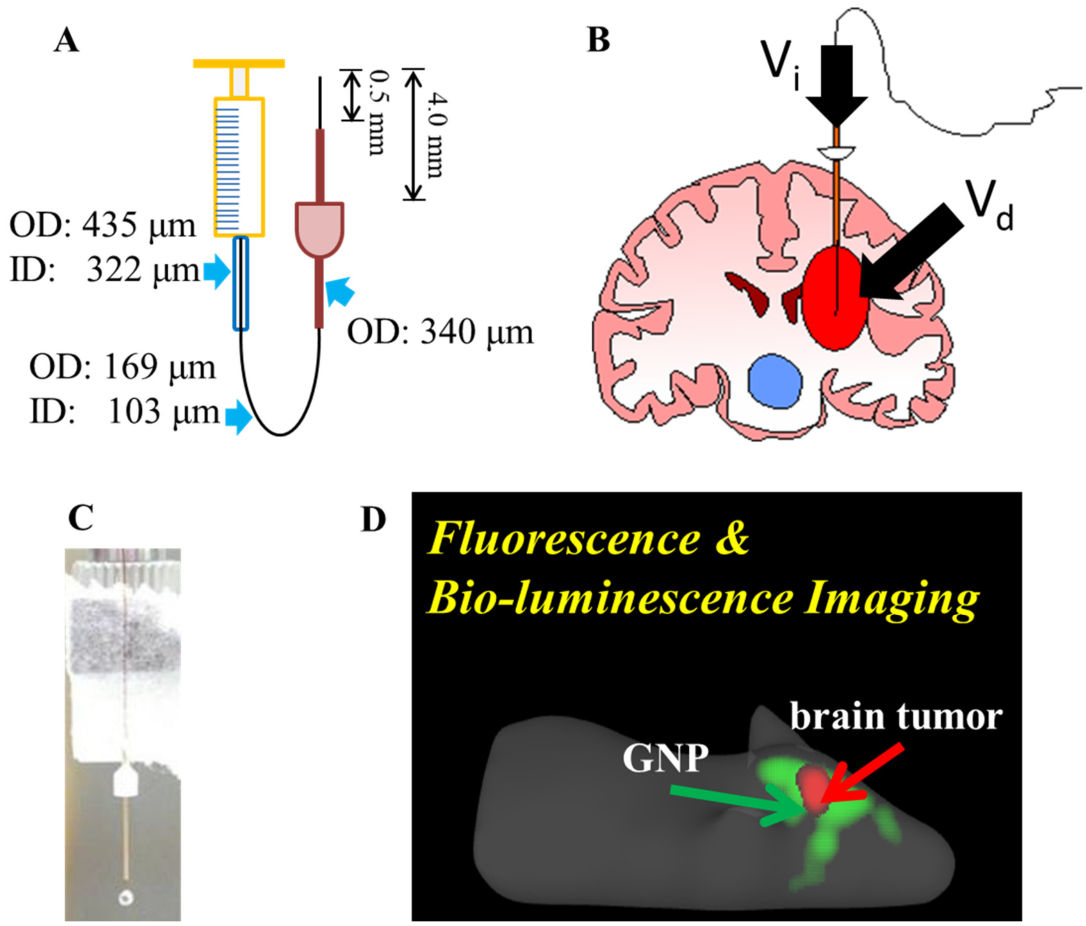

Figure S1. The convection-enhanced delivery (CED) was employed to deliver gold nanoparticles into an orthotopic brain tumor in mice. (A) the design of a homemade CED device, (B) a schematic diagram of drug delivery into the brain by CED, (C) the actual appearance of the CED device, and (D) the combination of bio-luminescence and fluorescence images to present the region of glioblastoma (red) and fluorescence-labeled GNP (green) in the brain of mice.

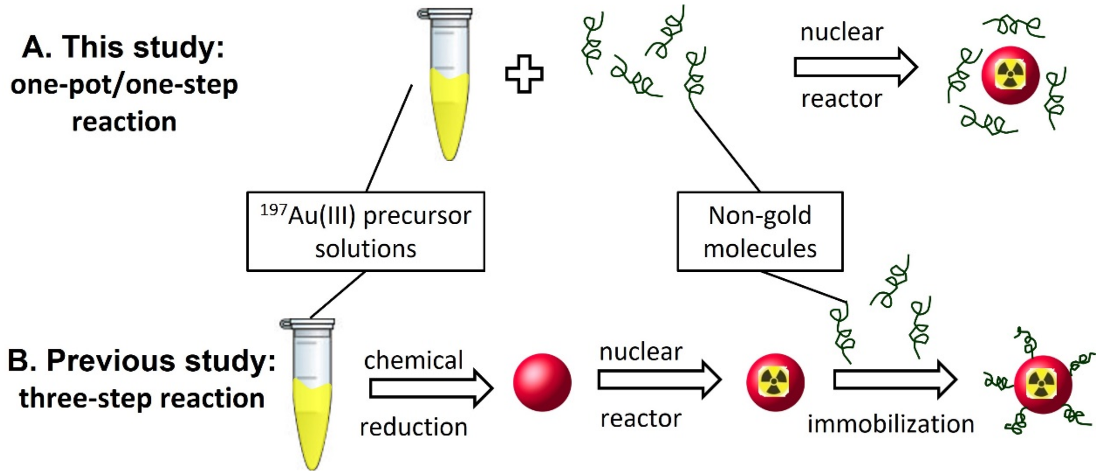

Figure S2. Two approaches are feasible to prepare radioactive gold nanoparticles. **(A)** In this study, the mixture of non-radioactive Au (III) precursor solution and polyethylene glycol was subjected to synthesize radioactive gold nanoparticles through the one-pot/one-step reaction in a nuclear reactor. **(B)** Three-step reaction published in the previous paper (*Analytical Chemistry* **87**, 601-608 (2015). doi: 10.1021/ac503260f) indicated non-radioactive gold nanoparticles synthesized first and then subjected to neutron irradiation in a nuclear reactor before immobilizing surface ligands onto radioactive gold nanoparticles.

Table S1. The chemical, physical, and radiochemical properties of RGNP in this study compared to that in a previous publication.

	**Chemical properties**		**Physical properties**			**Radiochemical properties**
Approaches	Reaction types	Molecules for surface modifications or protections	Particle size by TEM (nm)	Particle size by DLS (nm)	λ_{max} of UV-Vis (nm)	Major activated isotope in RGNP
This study	one-pot & one-step	PEG 6000, non-covalent bond	14.1±4.3 or 14.6±3.7 or 15.9±3.7	NA	519-521	^{198}Au
Analytical Chemistry **87**, 601-608 (2015) [39]	three-step	cPEG-SH 5000 or mPEG-SH 5000, covalent bond (Au-S)	13.2±1.0	14.4	519-520	^{198}Au
